# Prognosis in fertilisation rate and outcome in IVF cycles in patients with and without endometriosis: a population-based comparative cohort study with controls

**DOI:** 10.52054/FVVO.13.1.007

**Published:** 2021-03-31

**Authors:** J Metzemaekers, EER Lust, JPT Rhemrev, N Van Geloven, ARH Twijnstra, L Van Der Westerlaken, FW Jansen

**Affiliations:** Department of Gynaecology, Leiden University Medical Center, Leiden, the Netherlands; Department of Gynaecology, Haaglanden Medisch Centrum-Bronovo, Den Haag, the Netherlands; Department of Medical Statistics and Bioinformatics, Leiden University Medical Center, Leiden, the Netherlands; Department of Biomechanical Engineering, Delft University of Technology, Delft, the Netherlands.

**Keywords:** Endometriosis, IVF, fertilisation rate, zona pellucida

## Abstract

**Background:**

Subfertility occurs in 30-40% of endometriosis patients. Regarding the fertilisation rate with in vitro fertilisation (IVF) and endometriosis, conflicting data has been published. This study aimed to compare endometriosis patients to non-endometriosis cycles assessing fertilisation rates in IVF.

**Methods:**

A population-based cohort study was conducted at the Leiden University Medical Center. IVF cycles of endometriosis patients and controls (unexplained infertility and tubal pathology) were analysed. The main outcome measurement was fertilisation rate.

**Results:**

503 IVF cycles in total, 191 in the endometriosis group and 312 in the control. The mean fertilisation rate after IVF did not differ between both groups, 64.1%±25.5 versus 63.9%±24.8 (p=0.95) respectively, independent of age and r-ASRM classification. The median number of retrieved oocytes was lower in the endometriosis group (7.0 versus 8.0 respectively, p=0.19) and showed a significant difference when corrected for age (p=0.02). When divided into age groups, the statistical effect was only seen in the group of ≤ 35 years (p=0.04). In the age group ≤35, the endometriosis group also showed significantly more surgery on the internal reproductive organs compared to the control group (p<0.001). All other outcomes did not show significant differences.

**Conclusion:**

Similar fertilisation rates were found in endometriosis IVF cycles compared to controls. The oocyte retrieval was lower in the endometriosis group, however this effect was only significant in the age group ≤ 35 years. All other secondary outcomes did not show significant differences. In general, endometriosis patients with an IVF indication can be counselled positively regarding the chances of becoming pregnant, and do not need a different IVF approach.

## Introduction

Endometriosis is defined as the presence of endometrium-like tissue outside the uterus inducing chronic inflammation and the formation of adhesions (de Ziegler et al., 2010). The prevalence of the disease varies between 2 to 10% among the general female population of reproductive age, but rises up to 50% in infertile women (Eskenazi et al., 1997; Meuleman et al., 2009).

Subfertility is a major concern in women diagnosed with endometriosis and occurs in 30-40% (Young et al., 2016). In a review of Ziegler et al. (2010), it is stated that the cause of impaired fertility with endometriosis is multifactorial and affects the pelvic cavity, uterus and ovaries. In the pelvic cavity, endometriosis causes inflammatory changes in the peritoneal fluid which can affect the sperm- oocyte interaction. Also, endometriosis induces adhesion formation which negatively influences the delicate anatomy of the tube-ovarian-ovum pickup mechanism. Furthermore, the uterus is altered by endometriosis at the level of the endometrium itself, but also at the level of steroid synthesis influencing concentrations of oestrogen and progesterone. Last but not least, at the level of the ovaries, the function can be impaired due to endometriomas and consequently by repetitive surgery.

Regarding the quality of the oocytes, it is stated that chronic inflammation negatively impacts the oocytes in terms of clinical and biological outcomes (Sanchez et al., 2017). Oocyte quality is reflected in the ability to reach complete maturation and fertilisation. Considering the oocyte itself, one study even suggests that the zona pellucida of the oocyte might be thicker due to the toxic microenvironment with free radicals in patients with endometriosis (Goud et al., 2014). However, it is contradictory to note that the oocyte is possibly protecting the core from the toxic environment by alterations in the zona pellucida, but potentially also creates a barrier for the sperm cell to enter the oocyte. With this in mind, an interesting study was published by Komsky- Elbaz et al. (2013). This research group compared the fertilisation rates in endometriosis patients between sibling oocytes by dividing then into groups of intracytoplasmic sperm injection (ICSI) and IVF with normospermia semen. They found a significantly higher fertilisation rate in the ICSI group. From these results, they suggest that ICSI treatment in infertility patients with endometriosis might be a good clinical solution. This is in line with the thought that the zona pellucida is thicker in oocytes of patients with endometriosis, and that ICSI would be beneficial, since the ‘shell’ is bypassed with this approach.

Regarding the number of oocytes retrieved in IVF treatment in endometriosis, current studies show consistent and robust data about reduced oocytes retrieval (Senapati et al., 2016; Gonzalez- Comadran et al., 2017; Sanchez et al., 2017; Muteshi et al., 2018; Feichtinger et al., 2019). Based on these results, there is significant evidence that endometriosis patients harvest fewer oocytes (mostly one oocyte) compared to non-endometriosis patients. However, considering the fertilisation rate in patients with endometriosis undergoing IVF treatment, conflicting data has been published. Fertilisation rates range from reduced (Harb et al., 2013; Sanchez et al., 2017), to equal fertilisation rates (Senapati et al., 2016; Gonzalez-Comadran et al., 2017) and even increased rates (Muteshi et al., 2018). Based on these results, it can be stated that the evidence on the topic of fertilisation rate and endometriosis is still not conclusive, which can lead to different clinical approaches in women with endometriosis (e.g. ICSI).

Our study aims to inventory the fertilisation rates and outcomes of IVF cycles in endometriosis patients compared to their controls without endometriosis and to find characteristics that influence or predict the chances of success.

## Materials and Methods

### 

The study protocol was approved by the Medical Ethical Committee (G17.112). The IVF database, with prospectively collected data from cycles in the IVF laboratory of the Leiden University Medical Centre (LUMC) was analysed. The data was collected from both regional hospitals with transport service and the LUMC itself. So all IVF procedures took place at this laboratory.

Inclusion criteria in the control group were patients with tubal disease or unexplained infertility. Only patients with normospermia and fresh embryos (embryos from fresh oocytes, a procedure without cryopreservation) were included in both groups. Normospermia was defined regarding the World Health Organization (WHO), progressive motility of at least 32% and a minimal sperm concentration of 15 million per ml (Cooper et al., 2010). IVF cycles were categorised according to diagnosis. Endometriosis was staged as advised by the revised American Society Reproductive Medicine (r-ASRM, stage I - IV) (1997). If the stage was missing, the patient file was checked for detailed information on classification. The endometriosis diagnosis was either confirmed by laparoscopy or with imaging techniques such as ultrasound (US) or Magnetic Resonance Imaging (MRI). Tubal disease was confirmed by laparoscopy. Unexplained infertility refers to infertility in couples with apparently normal ovarian function, fallopian tubes, uterus, cervix and pelvis and with adequate coital frequency; and normal testicular function, genito- urinary anatomy and a normal ejaculate (Zegers- Hochschild et al., 2017). Only fresh and first cycles were included to reduce the potential bias that is induced by cryopreservation with its effect on the zona pellucida. Exclusion criteria consisted of cryopreserved cycles, ICSI cycles, no puncture due to low response and ovarian hyperstimulation syndrome (OHSS). Furthermore, oncology patients were also excluded. Regarding the controlled ovarian hyperstimulation protocol prior to the IVF procedure, the vast majority in this study period were treated with the short schedule agonist 150IU follitropin-alfa (Gonal-F) from day four. Endometriosis patients were not treated differently compared to the control group.

Baseline characteristics such as age, body mass index (BMI), smoking and alcohol consumption, primary or secondary infertility, previous surgery and pretreatment with gonadotropin-releasing hormone (GnRH) analogues were collected. Data on previous surgery was subdivided into 5 variables: abdominal surgery (including appendectomy, laparotomy, intestinal fistula surgery), reproductive surgery (including (laparoscopic) surgery of the fallopian tubes, uterus or ovaries), hysteroscopic surgery (including adhesiolysis, polypectomy, uterine septum surgery) or curettage, chromopertubation and no surgery.

The primary outcome was fertilisation rate. Fertilisation was considered normal when 2 pronuclei were present 16-20 hours after insemination. Two groups were identified: patients with clinically proven endometriosis and controls. The latter contained patients suitable for IVF without clinical signs of endometriosis.

Secondary outcomes included number of retrieved oocytes, number of embryo transfers (ET) (standard protocol, day 3 transfer), embryo morphology score (according to the number and size of blastomeres and the amount of fragmentation the embryos were assigned to four different quality scores: type 1: equal-sized blastomeres and no fragmentation; type 2: <20% fragmentation; type 3: 20-50% fragmentation; type 4: >50% fragmentation), pregnancy outcome (biochemical pregnancy: increasing bHCG>=50IU/l at 15 days after oocyte retrieval) and ongoing pregnancy (pregnancy with fetal heart rate).

A power calculation was made, based on the data of Komsky et al. (Komsky-Elbaz et al., 2013). To detect a 10 % difference in fertilization rate, which is considered clinically significant with 80% power and a two-sided α of 5%, 159 oocytes were needed in each group. This assumed a per group standard deviation of 31,9 and an analysis with a two-sided t-test with alpha 0.05.

### Statistical analysis

We used IBM SPSS version 25 for our analysis. Baseline and demographic characteristics were analysed with the Mann-Whitney U test and the Chi-Square test. As descriptive statistics, we used the mean and standard deviation for normally distributed variables or median and interquartile range for skewed variables. The independent T-test was used for the comparison of the mean fertilisation rate (normally distributed) and the Mann-Whitney U test for the number of retrieved oocytes (non-normal distribution) between endometriosis and the control group. With the analysis of covariance, the age- adjusted difference in mean fertilisation rate and the number of retrieved oocytes was calculated. Odds ratios (ORs) were estimated from univariate logistic regression models (ET, morphology, pregnancy test and ongoing pregnancy). Adjusted ORs were calculated after adjusting for age using multivariate logistic regression models. We performed correction for age since this is a well-known factor influencing fertilisation rates. Also, the relationship of BMI and primary or secondary infertility on fertilisation rates were analysed. We considered a p-value of < 0.05 as statistically significant.

## Results

The collection of data was undertaken respectively from 1998-2017 for endometriosis patients and 2010-2017 for the control group (Figure I flowchart inclusion). Of the 503 cycles, 191 (38%) were in the endometriosis group and 312 (62%) in the control group (42.9% tubal disease and 57.1% unexplained infertility). The longer inclusion period for endometriosis (1998-2010), added 31 extra endometriosis patients on the total endometriosis group (16.2% extra). This was done to get a more equal representation in cycles between endometriosis and the control group. No statistical difference was found between the fertilisation rate from cycles in the period between 1998-2010 and 2010-2017, p=0.69. Table I summarises the baseline characteristics of both groups. A notable significant difference was observed between the endometriosis and control group for median age (33.2 versus 36.1 respectively, p<0.001), primary infertility (68.6 % versus 43.6%, p<0.001), previous surgery on the reproductive organs (70.4% versus 30.8% respectively, p<0.001), chromopertubation (59.2% versus 39.9% respectively, p<0.001) and no surgery (9.1% versus 29.6% respectively, p<0.001). Regarding the endometriosis r-ASRM classification, 16% had stage I/II and 84% had stage III/IV. In 72 patients (38%) in the endometriosis group staging was not possible, because of no registered r-ASRM classification. No missing data regarding the primary outcome measures were found and the proportion of missing data for the secondary outcome measures was negligible (<5 %). We therefore performed a complete case analysis, no statistical corrections for the missing data was performed.

**Figure 1:**
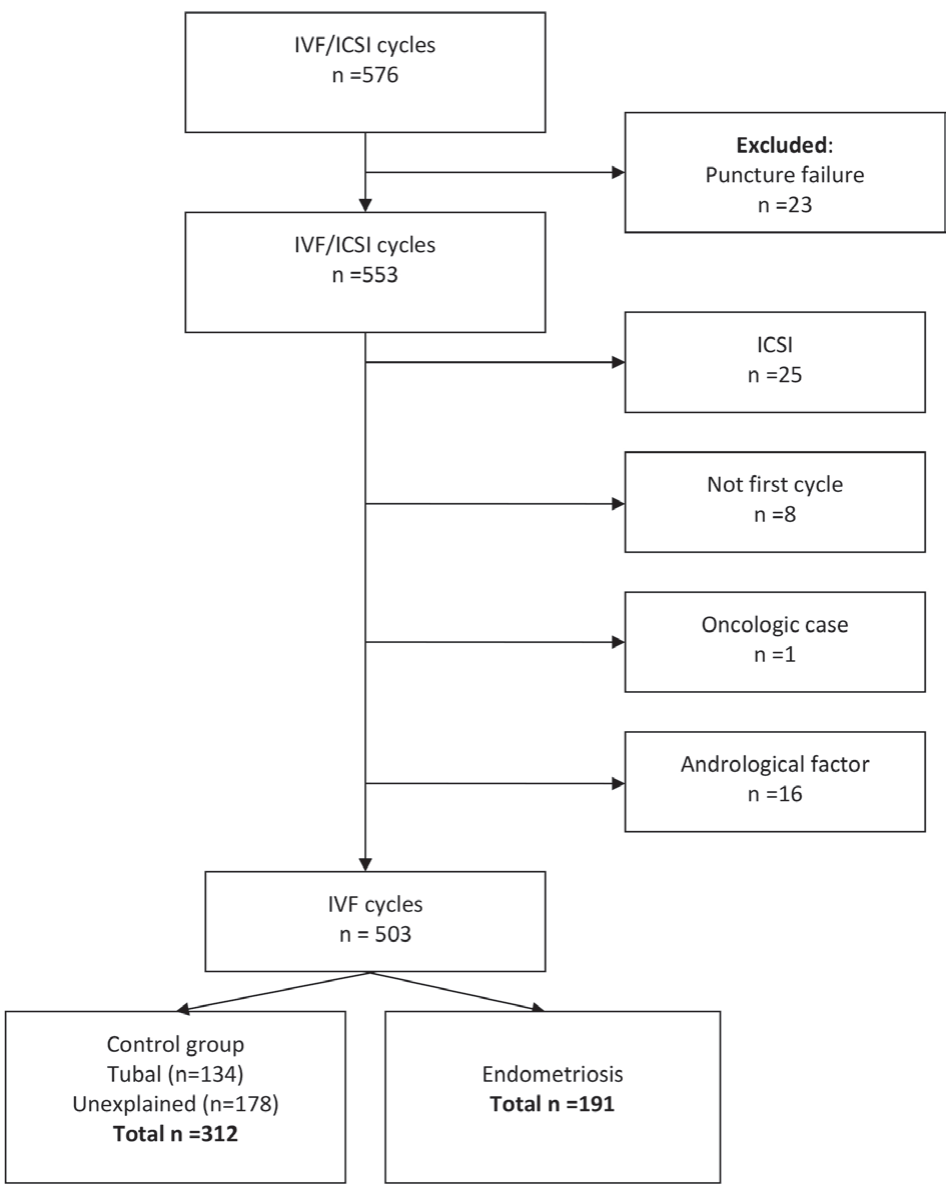
— Patient inclusion flowchart for IVF cycles.

**Table I:** Baseline characteristics of the patients.

Characteristics		Endometriosis (n=191)	Control^a^ (n=312)	p-value
Age at start IVF, median (Q1-Q3) years		33.2 (30.4-37.7)	36.1 (32.0-39.3)	<0.001
BMI, median ± (Q1-Q3) kg/m ^2^ ^b^		22.5 (20.6-25.7)	23.4 (21.5-26.0)	0.07
Smoking– n (%) ^c^		25 (15.6)	31 (13.7)	0.59
Drinking – n (%) ^d^		66 (42.6)	88 (40.6)	0.70
Infertility– n (%)				<0.001
	Primary	131 (68.6)	136 (43.6)	
	Secondary	60 (31.4)	176 (56.4)	
Previous surgery – n (%)				
	Abdominal surgery ^e^	29 (16.4)	30 (11.9)	0.18
	Reproductive surgery ^f^	126 (70.4)	78 (30.8)	<0.001
	Hysteroscopic surgery or curettage ^g^	27 (15.1)	31 (12.3)	0.40
	Chromopertubation ^f^	106 (59.2)	101 (39.9)	<0.001
	No surgery ^h^	8 (9.1)	55 (29.6)	<0.001
GnRH treatment– n (%) ^i^				0.24
	Lucrin	2 (1.1)	2 (0.8)	
	Synarel	27 (14.8)	44 (17.7)	
	Decapeptyl	121 (66.5)	157 (63.1)	
	Triptofem	4 (2.2)	0 (0.0)	
	Cetrotide	5 (2.7)	8 (3.2)	
	Orgalutran	4 (2.2)	3 (1.2)	
	Name unknown	19 (10.4)	33 (13.3)	
	No GnRH treatment	0 (0.0)	2 (0.8)	
Endometriosis classification ^j^				
	rASRM I/II – n (%)	19 (16)	-	
	rASRM III-IV – n (%)	99 (84)	-	

^a^ Defined as tubal disease + unexplained infertility as IVF indication; ^b^ Missing values: 55 (endometriosis), 139 (other); ^c^ Smoking at start of IVF treatment; Missing values: 31 (endometriosis), 85 (other); ^d^ Drinking at start of IVF treatment; Missing values: 36 (endometriosis), 95 (other); ^e^ Missing values: 14 (endometriosis), 59 (other); ^f^ Missing values: 12 (endometriosis), 59 (other); ^g^ Missing values: 7 (endometriosis), 60 (other); ^h^ Missing values: 103 (endometriosis), 126 (other); ^i^ Missing values: 9 (endometriosis), 63 (other); ^j^ The total number of observations was not 191 due to no r-ASRM classification in 72 patients.

The mean fertilisation rate after IVF did not differ between the two groups, 64.1±25.5 versus 63.9±24.8 (p=0.95) respectively (Table II). Furthermore, after correcting for age, there was no statistically significant difference in fertilisation rates. The age-adjusted mean difference was 1.2 with 95%CI (-5.8-3.4) (p=0.61) in fertilisation rate.

**Table II:** Primary outcome: Fertilisation rate.

		Endometriosis (n=189)	Control (n=307)	p-value	Adjusted mean diff. (95%CI)	p-value
Fertilisation rate (all) mean ± SD %		64.1±25.5 ^a^	63.9±24.8 ^b^	0.95	1.2 (-5.8-3.4)	0.61
	-age ≤ 35 ^d^	63.3±26.4	60.3±23.7	0.34		
	-age > 35 ^e^	65.1±66.5	66.5±25.3	0.69		
	-rASRM I/II ^f^	57.7±24.3		0.18		
	-rASRM III/IV ^f^	66.8±24.4				
	-proven endometriosis, missing stage	61.4±26.9				

^a^ The total number of observations was not 191 due to 0 oocytes retrieved in 2 patients; ^b^ The total number of observations was not 312 due to 0 oocytes retrieved in 5 patients; ^c^ Adjusted for age; ^d^ 114 cases in endometriosis group and 128 in control group; ^e^ 75 cases in endometriosis group and 179 cases in control group; ^f^ 19 cases in rASRM I/II group and 99 in the stage III/IV group, the significance is calculated between group rASRM I/II and III/IV.

No significant correlation was found between the effect of BMI and primary/secondary infertility at the level of fertilisation rates. Also no significant difference (p=0.18) between r-ASRM stage I/II compared to stage III/IV regarding the fertilisation rate was found. And no statistical difference was found in the fertilisation rates between the surgical staged endometriosis group (rASRM I- IV) and the group without an rASRM stage (p=0.30).

For retrieved oocytes, no significant difference was observed between the endometriosis and the control group (Table III), the median number was 7 (5-10) versus 8 (4-12) respectively (p=0.19). However, we found a significantly lower mean number of retrieved oocytes in endometriosis patients compared to the control patients when correcting for age (age-adjusted mean difference -1.1, 95%CI=- 2.0- -0.2, p=0.02). When we divided the oocyte retrieval into two age groups, it was shown that in the group of ≤ 35 years, the endometriosis group had a significant lower oocyte retrieval (median of 8) compared to the control (median of 10) p=0.04. This effect was not seen in the age group of >35. In the age group ≤ 35 years, the endometriosis group had significantly more surgery on internal reproductive organs (71.8%) compared to the control group (35.2%) (p<0.001).

**Table III:** Secondary outcome measures.

		Endometriosis (n=191)	Control (n=312)	Univariable Odds ratio (95%CI)	p-value	Multivariable ^*^ Adjusted Odds ratio (95%CI)	p-value
Number of retrieved oocytes, median (Q1-Q3)		7 (5-10)	8 (4-12)		0.19		
	≤ 35 years	8 (5-11)	10 (6-13)		0.04		
	> 35 years	6 (4-9)	7 (4-11)		0.32		
No. of embryo transfers (ET) – n (%) ^a^							
	SET	144 (80.9)	245 (82.5)	0.90 (0.56-1.45)	0.66	0.61 (0.36-1.03)	0.06
	DET	34 (19.1)	52 (17.5)				
Embryo morphology score – n (%) ^b^							
Embryo 1							
	type 1	73 (40.8)	143 (48.1)	0.74 (0.51-1.08)	0.12	0.70 (0.48-1.03)	0.07
	type 2/3	106 (59.2)	154 (51.9)				
Embryo 2							
	type 1	9 (26.5)	15 (28.8)	0.88 (0.34-2.34)	0.81	0.91 (0.33-2.52)	0.86
	type 2/3	25 (73.5)	37 (71.2)				
Pregnancy test- n (%) ^c^							
	positive	68 (38.2)	86 (29.0)	1.52 (1.02-2.25)	0.04	1.37 (0.92-2.05)	0.12
	negative	110 (61.8)	211 (71.0)				
Ongoing pregnancy ^d^							
	yes	52 (29.4)	64 (21.8)	1.49 (0.97-2.28)	0.07	1.30 (0.84-2.02)	0.24
	no	125 (70.6)	229 (78.2)				

SET=Single embryo transfer, DET=Double embryo transfer, CI= confidence interval.

^*^ adjusted model controls for age; ^a^ Not applicable to all patients, because of no embryo transfer in 13 patients, (endometriosis), 15 patients (other); ^b^ Morphology score 1-best, 4-worst. Morphology score performed on day 2; ^c^ The pregnancy test was performed two weeks after ET. Missing values: 1 (endometriosis); ^d^ Defined as pregnancy ≥ 12 weeks. Missing values: 2 (endometriosis), 4 (other).

The number of embryo transfers in endometriosis versus control cycles did not show significant differences. Respectively, the single embryo transfer number was 144 (80.9%) versus 245 (82.5%) for the control, the double embryo transfer number was 34 (19.1%) versus 52 (17.5%) (p=0.66).

The endometriosis group had similar embryo morphology scores in the first embryos compared to the control group: type 1 73 (40.8%) versus 143 (48.1%), type 2-3 106 (59.2%) versus 154 (51.9%) (p=0.12). Also, no statistical difference was found in the embryo morphology scores of the second embryos in case of a double embryo.

Patients with endometriosis had more often a positive pregnancy test compared to the control group (38.2% versus 29.0% respectively, OR 1.52 95%CI (1.02-2.25) (p=0.04). This significance was lost when we adjusted for age (age adjusted OR=1.37 95%CI (0.92-2.05) (p=0.12)). No statistical difference was observed in ongoing pregnancy rates: 52 (29.4%) in the endometriosis group versus 64 (21.8%) in the control (OR=1.49 (0.97-2.28) p=0.07; adjusted OR 1.30 (0.84-2.02) (p= 0.24).

## Discussion

We found comparable fertilisation rates in endometriosis patients compared to their controls, independent of age and r-ASRM stage. In the literature, there is inconsistency regarding fertilisation rates in endometriosis IVF cycles. These outcomes range from lower, equal and even higher percentages. The results from our study support the current studies with equal fertilisation rates (Senapati et al., 2016; Gonzalez-Comadran et al., 2017). This is seen in Table IV, where we present a concise overview of important studies with fertilisation rates in endometriosis and other IVF outcomes.

**Table IV:** Overview of important studies with Fertilisation Rate (FR) outcomes in endometriosis and controls, compared to our study results.

Study		Year	N	Type of study	FR	Oocyte retrieval	Morphology	Preg. test	Ongoing preg.
Muteshi et al.		2018	1.268	retrospective cohort	higher	lower		lower	equal
Sanchez et al.		2017	-	retrospective cohort	lower	lower	inferior		
Gonzales et al.		2017	22.416	retrospective cohort					
	≤ 35 years				equal	lower			equal
	> 35 years			retrospective cohort	equal	lower			equal
Senapati et al.		2016	347.185	review					
Harb et al.		2013	-						
	stage I/II				lower				equal
	stage III/IV				equal				lower
Our study		2020	503	retrospective cohort	equal	lower	equal	equal	equal
	≤ 35 year				equal	lower			
	> 35 years				equal	equal			

lower or inferior = red and better or higher = green N = amount of inclusions

Additionally, we also found that the endometriosis patients facing infertility started earlier when proceeding with IVF. Furthermore, the endometriosis group had a higher rate of primary infertility and underwent more surgery on internal reproductive organs before starting the IVF procedure. The endometriosis group (age ≤ 35 years) had significantly lower oocyte retrieval compared to the control group, this effect was not seen in the age group older than 35 years. Finally, the morphology of the embryos and the ongoing pregnancies were similar for both groups. Therefore we can carefully conclude that endometriosis patients do not need a different clinical IVF approach compared to patients without endometriosis. Furthermore, the idea that ICSI treatment would be beneficial for endometriosis patients due to lower fertilisation rates is not supported by our findings.

Our results show that the fertility outcomes of endometriosis patients has a more positive outlook, since we found that these patients have the same fertility chances at IVF compared to their controls, without endometriosis. The theory that oocytes of endometriosis patients might have a thicker zona pellucida is, therefore, less likely if we look at the level of fertilisation. It might be true that the zona pellucida is thicker in these patients (Goud et al., 2014), but probably without any clinical consequences on the fertilisation rate. The study that compared zona pellucida dissolution timing in endometriosis and control patients found a longer dissolution time in the endometriosis group (133.8 ± 9.4 s vs. 90.5 ± 5.8 s). Translating these findings of zona pellucida dissolution differences in endometriosis compared to controls apparently does not influence the fertilisation rate in IVF treatment.

The lower oocyte retrieval highlighted in our study is supported by others also (Senapati et al., 2016; Gonzalez-Comadran et al., 2017; Sanchez et al., 2017; Muteshi et al., 2018; Feichtinger et al., 2019). Combining their studies with our results, we can conclude that endometriosis patients retrieve significantly lower oocytes compared to their controls. However, we found that the overall difference is often not more than one or two oocytes. It is therefore arguable if this is clinically relevant. The recent European Society of Human Reproduction and Embryology guideline (Bosch et al., 2020) states that “We still need to see evidence that a few oocytes more or less will make the desired or feared difference in terms of live birth rates”. This shows that it is still not clear whether one or two oocytes extra will make the difference.

The minimal oocyte retrieval difference between endometriosis and control patients might not have a relevant clinical difference, albeit the literature shows that with increasing age, the number of retrieved oocytes more than halved from age 25 to 41 years (Boer et al., 2004). So, age is a significant prognostic factor as expected, meaning that the chances of IVF are better when sub- and infertile couples start on time. In this context, on time means women who have not yet become reproductively old ~36 or younger (Leridon, 2004).

From our data, we carefully speculate that the causal accent of fertility problems in women with endometriosis might be more due to repetitive surgery and subsequent adhesions formation, rather than on the level of poor quality of oocytes due to a chronic inflammation process. Others also found this negative impact of surgery on ovarian function and fertility (Raffi et al., 2012; Chiang et al., 2015; Kalra et al., 2016). While most studies reporting a lower oocyte retrieval did not report previous surgery in their baseline characteristics, we found a significantly lower oocyte retrieval in the endometriosis group who had more surgery. Therefore it might be advisable to be more reticent in the surgical approach (internal reproductive organs) towards women with a future pregnancy wish, to preserve ovarian tissue as much as possible and therefore the oocyte reserve. This is further supported by literature whereby healthy ovarian tissue was found in women who underwent surgical excision of endometriomas (Gupta et al., 2006; Muzii et al., 2007).

Regarding our other secondary baseline outcomes; embryo morphology score, pregnancy test and ongoing pregnancy, we found no significant differences between groups. Contrary to our results, the systematic review of Sanchez et al. (2017) reported that altered morphology is present in the oocytes of patients with endometriosis. We did not power our study on the secondary outcomes, so it remains difficult to draw a solid conclusion from our data about morphology, however looking at our database we cautiously conclude that there was no difference.

Looking at the pregnancy test and ongoing pregnancy, we did not find any differences between both groups in the age-adjusted analysis. Our results, of ongoing pregnancy, are supported by several recent studies (Muteshi et al., 2018; Feichtinger et al., 2019).

It should be noted that the majority of the patients in our study were pretreated with GnRH agonists. It is equally recommended to use agonist or antagonists for expected poor responders (endometriosis patients) (Bosch et al., 2020). However, expected high- and normal responders are, at the moment, preferably pretreated with GnRH antagonists. For the generalisability of our findings, it should be kept in mind that our study mainly consisted of GnRH agonists.

A strength of our study is that we only used fresh embryos, to reduce the possible impaired effect of cryopreservation on the quality. We believed in the importance of using fresh cycles since cryopreservation and thawing of embryos potentially harm the zona pellucida and therefore could bias the results (Fabbri, 2006). It would be interesting to perform future research on specifically cryopreserved cycles to gain insight into this phenomenon. Another strength of our study is that we looked at a variety of IVF outcomes; fertilisation rate, oocyte retrieval, morphology, pregnancy test and ongoing pregnancies. This makes our study more robust in its variety of outcomes.

A limitation of our study is that the time of inclusion of cycles in the endometriosis group ranges from 1998-2017 and in the control group from 2010-2017. This difference occurred because the endometriosis patients were less represented in our database over the years compared to the control group. However, we were aware of the fact that this could potentially introduce bias, especially since IVF protocols have changed to improve fertilisation techniques. Therefore, we performed an analysis on the data with and without the cohort from 1998- 2010 in the endometriosis group. No significant effect was found on the results. Furthermore, the fertilisation rate stayed the same over time. If this issue of bias would have occurred, it could be expected that patients treated with ‘older’ IVF techniques would have resulted in poorer outcomes in the endometriosis group. However, this did not occur in time.

A second limitation is the missing data on endometriosis classification. This is a well- recognised problem in the literature (clinic and research field) and therefore the World Endometriosis Society (WES) recommends classifying each endometriosis patient according to the r-ASRM classification (stage I-IV) (Johnson NP et al., 2017). In our study, we had to deal with a significant amount of missing r-ASRM stages. However, there was no doubt about the indication of IVF: endometriosis. This diagnosis was checked in retrospect in the patient’s record to ultimately support this factor from prospectively obtained data. From now on all our clinicians provide all patients with r-ASRM classification, to prevent research problems like this in future.

## Conclusion

Our research highlighted that fertilisation rates are similar in endometriosis and control patients, independent of age and r-ASRM stage. Regarding pregnancy test, ongoing pregnancies and morphology we did not find any differences compared to the controls. Only oocyte retrieval was significantly lower in the endometriosis group compared to controls in the age group of ≤ 35 years. The endometriosis group also showed significantly more surgery on the internal reproductive organs, this could be one explanation for the reduced oocyte retrieval. This suggests that a more reticent surgical approach might be favorable for endometriosis patients. Regarding endometriosis patient counselling, we can conclude that they have overall equal IVF chances compared to non-endometriosis patients and do not need a different IVF approach.

## References

[1] Boer E, Tonkelaar I, Burger C (2004). The number of retrieved oocytes does not decrease during consecutive gonadotrophin-stimulated IVF cycles.. Hum Reprod.

[2] Bosch E, Broer S, Griesinger G (2020). ESHRE guideline: ovarian stimulation for IVF/ICSI.. Hum Reprod Open.

[3] Chiang HJ, Lin PY, Huang FJ (2015). The impact of previous ovarian surgery on ovarian reserve in patients with endometriosis.. BMC Womens Health.

[4] Cooper TG, Noonan E, von Eckardstein S (2010). World Health Organization reference values for human semen characteristics.. Hum Reprod.

[5] De Ziegler D, Borghese B, Chapron C (2010). Endometriosis and infertility: pathophysiology and management.. Lancet.

[6] Eskenazi B, Warner ML (1997). Epidemiology of endometriosis.. Obstet Gynecol.

[7] Fabbri R (2006). Cryopreservation of human oocytes and ovarian tissue.. Cell Tissue Bank.

[8] Feichtinger M, Nordenhok E, Olofsson JI (2019). Endometriosis and cumulative live birth rate after fresh and frozen IVF cycles with single embryo transfer in young women: no impact beyond reduced ovarian sensitivity-a case control study.. J Assist Reprod Genet.

[9] Gonzalez-Comadran M, Schwarze JE, Zegers-Hochschild F (2017). The impact of endometriosis on the outcome of Assisted Reproductive Technology.. Reprod Biol Endocrinol.

[10] Goud PT, Goud AP, Joshi N (2014). Dynamics of nitric oxide, altered follicular microenvironment, and oocyte quality in women with endometriosis. Fertil Steril.

[11] Gupta S, Agarwal A, Agarwal R (2006). Impact of ovarian endometrioma on assisted reproduction outcomes.. Reprod Biomed.

[12] Harb HM, Gallos ID, Chu J (2013). The effect of endometriosis on in vitro fertilisation outcome: a systematic review and meta-analysis.. BJOG.

[13] Johnson NP, Hummelshoj L, Adamson GD (2017). World Endometriosis Society consensus on the classification of endometriosis.. Hum Reprod.

[14] Kalra GS, Campbell S, Nargund G (2016). Ovarian reserve may be compromised after adnexal surgery: Are we sufficiently fertility- focused in our surgical training?. Facts Views Vis Obgyn.

[15] Komsky-Elbaz A, Raziel A, Friedler S (2013). Conventional IVF versus ICSI in sibling oocytes from couples with endometriosis and normozoospermic semen.. J Assist Reprod Genet.

[16] Leridon H (2004). Can assisted reproduction technology compensate for the natural decline in fertility with age? A model assessment.. Hum Reprod.

[17] Meuleman C, Vandenabeele B, Fieuws S (2009). High prevalence of endometriosis in infertile women with normal ovulation and normospermic partners.. Fertil Steril.

[18] Muteshi CM, Ohuma EO, Child T (2018). The effect of endometriosis on live birth rate and other reproductive outcomes in ART cycles: a cohort study.. Hum Reprod Open 2018.

[19] Muzii L, Bianchi A, Bellati F (2007). Histologic analysis of endometriomas: what the surgeon needs to know.. Fertil Steril.

[20] Raffi F, Metwally M, Amer S (2012). The impact of excision of ovarian endometrioma on ovarian reserve: a systematic review and meta-analysis.. J Clin Endocrinol Metab.

[21] (1996). Revised American Society for Reproductive Medicine classification of endometriosis: 1996. Fertil Steril.

[22] Sanchez AM, Vanni VS, Bartiromo L (2017). Is the oocyte quality affected by endometriosis? A review of the literature.. J Ovarian Res.

[23] Senapati S, Sammel MD, Morse C (2016). Impact of endometriosis on in vitro fertilization outcomes: an evaluation of the Society for Assisted Reproductive Technologies Database. Fertil Steril.

[24] Young K, Fisher J, Kirkman M (2016). Endometriosis and fertility: women’s accounts of healthcare.. Hum Reprod.

[25] Zegers-Hochschild F, Adamson GD, Dyer S (2017). The International Glossary on Infertility and Fertility Care, 2017.. Fertil Steril.

